# Superficial spreading cervical squamous cell carcinoma *in situ* involving the endometrium: a case report and review of the literature

**DOI:** 10.1186/s13256-022-03433-4

**Published:** 2022-05-20

**Authors:** Javier Martín-Vallejo, Juan B. Laforga, Patricia Molina-Bellido, Pedro A. Clemente-Pérez

**Affiliations:** 1 Department of Obstetrics and Gynecology, Hospital de Denia, Avenida Marina alta, s/n, 03700 Denia, Alicante Spain; 2 Department of Pathology, Hospital de Denia, Avenida Marina alta, s/n, 03700 Denia, Alicante Spain

**Keywords:** Cervical squamous cell carcinoma, Endometrium, Papillomavirus, Superficial spreading, Case report

## Abstract

**Background:**

The spread of cervical squamous cell carcinoma to the inner surface of the uterus with replacement of the endometrium is rare. Continuity of the lesion must be demonstrated to confirm superficial spread and rule out concomitant endometrial cancer.

**Case presentation:**

We present the case of a 66-year-old white woman with superficial spreading squamous cell carcinoma of the cervix that involved the endometrium. Her relevant past history included conization of the cervix to treat cervical intraepithelial neoplasia III with positive margins. She subsequently had three negative cervical vaginal cytology results, each with a positive high-risk human papillomavirus test. Transvaginal ultrasound showed occupation of the entire uterine cavity by dense material consistent with pyometra in addition to myometrial thinning due to tension and cervical dilation. The patient presented with greenish vaginal discharge of 3 months’ duration. The cervix was not visible during speculum examination. Access for endometrial sampling was not possible, raising suspicion of post-conization cervical stenosis. The patient was treated with laparoscopic hysterectomy with double adnexectomy. Histologic examination showed superficial squamous cell carcinoma invading the cervix to a depth of 2.8 mm; superficial spreading squamous cell carcinoma *in situ* was also observed in the lower uterine segment and endometrium. The patient was free of symptoms 12 months after surgery.

**Conclusions:**

Squamous cell carcinoma of the cervix with superficial spread to the endometrium is not included in the 2020 (fifth edition) World Health Organization Classification of Female Genital Tract Tumors or the 2018 International Federation of Gynecology and Obstetrics cervical cancer staging system. More clinical cases are needed to identify other prognostic factors and inform clinical practice guidelines on the management of this disease.

## Background

Squamous cell carcinoma (SCC) accounts for approximately 80% of all cervical cancers and is the fourth most common cancer in women worldwide [1]. Superficial spreading SCC is a form of cervical SCC that extends superficially to the inner surface of the uterus, replacing the endometrium. There are insufficient data to compare superficial spreading SCC of the cervix with other types of cervical cancer. More clinical cases are needed to identify additional prognostic factors and inform clinical practice guidelines on the management of this disease.

## Case presentation

**Fig.1:**
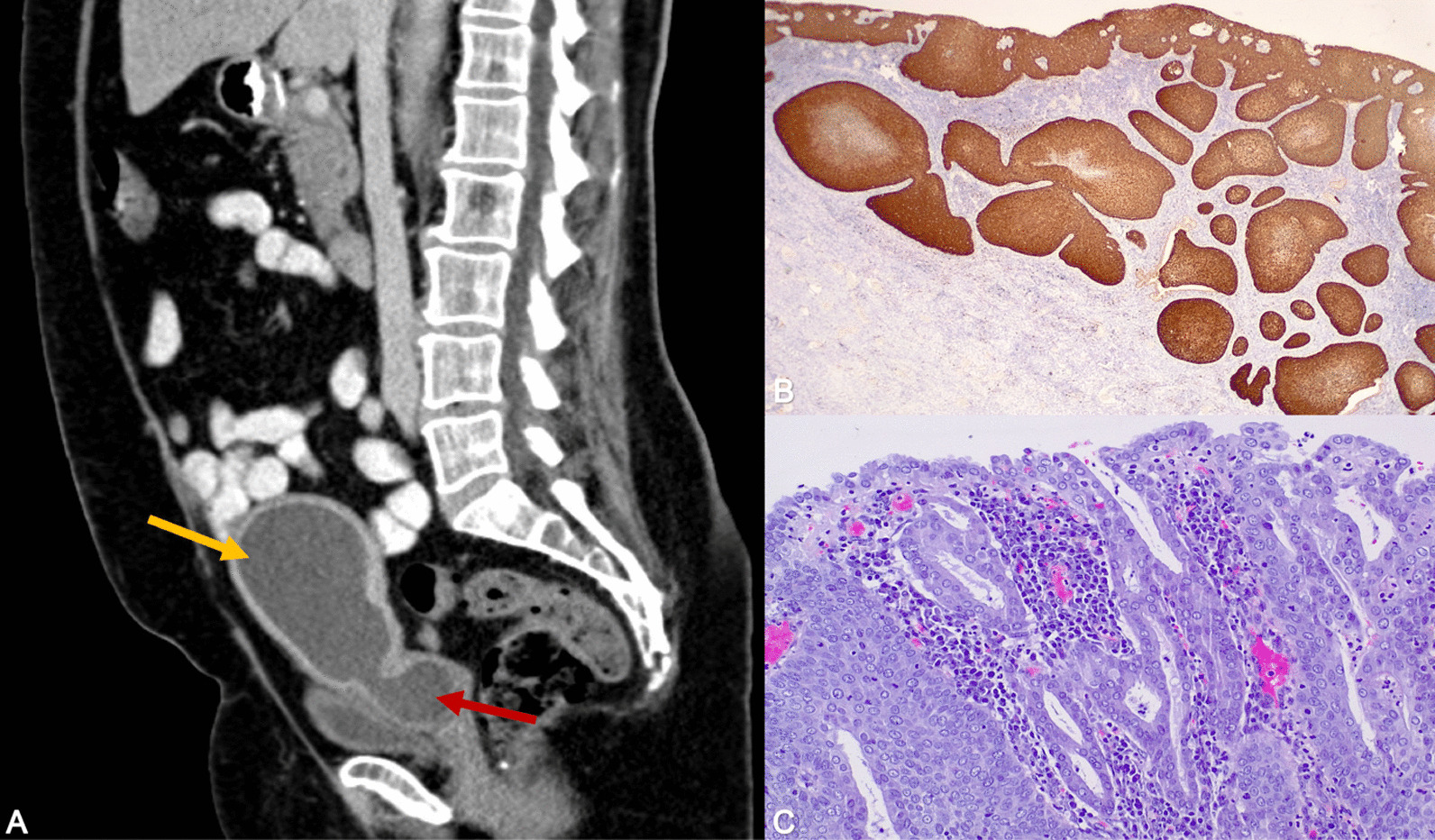
**A** Contrast-enhanced computed tomography scan. Marked distension of the uterine cavity (yellow arrow) and cervical canal (red arrow). Presence of dense material consistent with pyometra. **B** Squamous cell carcinoma with superficial invasion (2.8 mm). Staining for p16 ×250. **C** Note the large number of endometrial glands in the tumor. Hematoxylin–eosin ×100

The patient was a 66-year-old white woman who had had six pregnancies (four live births via Cesarean delivery and two miscarriages) and reached menopause at age 51 years. She did not smoke tobacco or drink alcohol. She had a history of hypertension and dyslipidemia and is currently taking enalapril and atorvastatin. She has no remarkable family history. In her country of origin, she had undergone conization of the cervix to treat cervical intraepithelial neoplasia (CIN) III with positive margins in 2011. She did not attend any follow-up appointments. In 2014, she presented for clinical evaluation and had a normal cytology result, but tested positive for high-risk human papillomavirus (HR-HPV); colposcopic examination showed no apparent lesions. The patient visited our clinic for the first time in 2015 and underwent cervical and vaginal cytology. She was asymptomatic at the time. The sample was satisfactory for analysis and tested negative for atypical cells and positive for HR-HPV 16. Co-testing was scheduled for a year later, but the patient did not attend the appointment and was lost to follow-up. She returned in 2020, presenting with greenish vaginal discharge of 3 months’ duration. The cervix was not visible during speculum examination; a point-like orifice consistent with the cervical canal was observed towards the right of the vaginal fornix. It was not possible to gain access for endometrial sampling, and post-conization cervical stenosis was suspected. Transvaginal ultrasound showed occupation of the entire uterine cavity by dense material consistent with pyometra, in addition to myometrial thinning due to tension and cervical dilation. Cervical and vaginal cytology was negative for atypical cells and positive for HPV 16. Contrast-enhanced computed tomography of the abdomen and pelvis (Fig. 1A) confirmed the presence of pyometra (139 mm × 70 mm × 61 mm). The staging study was negative. The patient was treated with laparoscopic hysterectomy with double adnexectomy. Examination of the abdominal cavity showed no abnormal findings. The physical and neurological examination on admission was normal. Upon arrival, her vital signs were blood pressure 130/80 mmHg, pulse rate 80 beats per minute, respiratory rate 20 breaths per minute, and body temperature 36.8 °C. The results of routine blood tests showed a normal blood cell count; hemoglobin (Hgb) 12.1 g/dl, leukocytes (10 × 10^3^/μl), neutrophils: 7 × 10^9^/L, platelets 250 × 10^9^/L, negative C-reactive protein (CRP; < 0.5 mg/L). Screening for hepatitis B, hepatitis C, and human immunodeficiency virus (HIV) serologies were done and found to be negative. Furthermore, liver enzymes, coagulogram, urea and creatinine, and acid–base status of the blood were normal. During admission, intravenous analgesia was prescribed with 1 g of paracetamol every 8 hours. The patient was discharged on clinical day 1 and hemodynamically stable. Oral antiinflammatories were prescribed as needed, and she did not require readmission. Follow-up visits were every 4 months. The patient was symptom free 12 months after surgery.

Histologic examination showed superficial SCC invading the cervix to a depth of 2.8 mm and occupying all quadrants; p16 staining was positive. The diagnosis was HPV-associated SCC. Superficial spreading SCC *in situ* was also observed in the lower uterine segment and endometrium (Fig. 1B, C). There were no signs of lymphovascular invasion or infiltration of the fallopian tubes or ovaries. SCC of the cervix with superficial spread to the endometrium is rare. It is not included in the 2020 (fifth edition) World Health Organization (WHO) Classification of Female Genital Tract Tumors or the 2018 FIGO (International Federation of Gynecology and Obstetrics) cervical cancer staging system. Nonetheless, we consider that the patient had stage IA1 (FIGO 2018) pT1a1 (American Joint Committee on Cancer 2018) disease, and as such the treatment was sufficient.

## Discussion and conclusions

We present the case of a woman with superficial spreading SCC of the cervix that involved the endometrium. We include ultrasound and microscopy images to perfectly illustrate the clinical case. We review the literature based on 54 cases retrieved by a keyword search in PubMed and Medline in Table 1. This review is the largest to date on this topic. Superficial spreading SCC of the cervix occurs mainly in menopausal women with history of cervical conization. The most common clinical presentation is vaginal bleeding and pyometra.

**Table 1:** Reported cases of superficial spreading squamous cell carcinoma of uterine cervix involving the endometrium and upper genital tract

Author (year)	Case	Age	Clinical presentation	Cervical lesion	Extension of lesion	Follow-up (months)	Outcome
Langley *et al.* (1956) [16]	1	64	NA	Invasive	Endometrium and bilateral Fallopian tubes	0	DED (postsurgical)
Friedell *et al.* (1958) [17]	2	55	NA	Invasive	Endometrium (*in situ*)	36	NED
		56	NA	Invasive	Endometrium (*in situ*)	36	NED
Brocheriou *et al.* (1963) [18]	1	63	Pyometra	Invasive	Endometrium (*in situ*)	NA	NA
Kairys *et al.* (1964) [19]	1	57	Pyometra	Invasive	Endometrium (*in situ*)	NA	NA
Delattre *et al.* (1965) [20]	1	66	Cervical stenosis and pyometra	Invasive	Endometrium (*in situ*)	12	NED
Salm *et al.* (1967) [21]	3	67	Pyometra	Carcinoma *in situ*	Endometrium (*in situ*)	66	NED
		44	NA	Carcinoma *in situ*	Endometrium (*in situ*)	3	NED
		70	NA	Invasive	Endometrium and vagina (*in situ*)	180	NED
Weill *et al.* (1968) [22]	1	69	Pyometra	Carcinoma *in situ*	Endometrium and left fallopian tube (*in situ*)	NA	NA
Hallgímson *et al.* (1969) [23]	1	54	Pyometra	Carcinoma *in situ*	Endometrium and bilateral fallopian tubes (*in situ*)	NA	NA
Ferenczy *et al.* (1971) [24]	1	53	Abnormal pap smears	Carcinoma *in situ*	Endometrium (*in situ*)	84	NED
Quizilbash *et al.* (1975) [25]	1	63	Vaginal bleeding	Invasive	Endometrium and bilateral fallopian tubes (*in situ*)		
Kamalian *et al.* (1977) [26]	1	55	Vaginal bleeding	Invasive	Endometrium (*in situ*)	NA	NA
Schmitt *et al.* (1977) [27]	4	59	Abnormal pap smears	Carcinoma *in situ*	Endometrium (*in situ*)	NA	NA
		65	Abnormal pap smears	Invasive	Endometrium (invasive)	NA	NA
		58	Cervical stenosis	Invasive	Endometrium (invasive)	NA	NA
		52	Vaginal bleeding	Invasive	Endometrium (*in situ*)	NA	NA
Kanbour *et al.* (1978) [10]	5	66	Pyometra	Invasive	Endometrium (*in situ*, microinvasive foci)	4	DOD
		58	Pyometra	Invasive	Endometrium (*in situ*)	132	NED
		53	Cervical stenosis and pyometra	Invasive	Endometrium (*in situ*)	54	DOD
		61	Pyometra	Invasive	Endometrium (*in situ*)	48	NED
		54	Pyometra	Invasive	Endometrium (invasive)	42	NED
Gupta *et al.* (1979) [15]	1	67	Vaginal bleeding	Carcinoma *in situ*	Endometrium (*in situ*)	NA	NA
Punnone *et al.* (1979) [28]	1	64	Abnormal pap smears	Invasive	Endometrium and right fallopian tube (*in situ*)	NA	NA
Sandhyamani *et al.* (1983) [29]	1	NA	NA	Invasive	Endometrium, fallopian tube, and vagina (*in situ*)	NA	NA
Daniele *et al.* (1985) [30]	1	NA	NA	Carcinoma *in situ*	Endometrium (*in situ*)	NA	NA
Motoyama *et al.* (1988) [31]	1	59	Vaginal bleeding, lower abdominal mass	Invasive	Endometrium, left fallopian tube, left ovarian and pelvic lymph nodes (invasive)	9	DOD
Teixera *et al.* (1991) [32]	1	64	Pyometra	Carcinoma *in situ*	Endometrium (invasive), pelvic lymph nodes (invasive)	NA	NA
Razquin *et al.* (1993) [33]	1	52	Cervical stenosis and pyometra	Carcinoma *in situ*	Endometrium and right fallopian tube (*in situ*)	72	NED
Pins *et al.* (1997) [34]	1	55	Abnormal pap smears	Carcinoma *in situ*	Endometrium (*in situ*), bilateral tubes (*in situ*), bilateral ovaries (invasive)	42	NED
Kushima *et al.* (2004) [3]	5	68	Genital discharge	Carcinoma *in situ*	Endometrium (*in situ*, focal microinvasive), left fallopian tube (invasive), left ovary (invasive)	54	NED
		58	NA	Carcinoma *in situ*	Endometrium (single focus of microinvasion, <1 mm in depth)	NA	NA
		72	Hematometra	Carcinoma *in situ*	Endometrium (*in situ*)	30	NED
		78	Vaginal bleeding	Invasive	Endometrium (invasive), vagina (*in situ*)	NA	NA
		59	Vaginal bleeding, lower abdominal mass	Invasive	Endometrium (*in situ* with endometrial stroma sarcoma), left fallopian tube, left ovary, vagina (*in situ*), vulva (*in situ*)	NA	NA
Tan *et al.* (2004) [8]	1	70	Vaginal bleeding	Microinvasive	Endometrium (*in situ*)	6	NED
Agashe *et al.* (2007) [35]	1	NA	NA	Carcinoma *in situ*	Endometrim, bilateral fallopian tubes and ovaries (*in situ*)	NA	NA
Alder *et al.* (2007) [36]	1	59	Lower abdominal mass	Invasive	Endometrium (invasive)	NA	NA
Gungor *et al.* (2011) [7]	1	53	Vaginal bleeding	Invasive	Endometrium (*in situ*, focal myometrial involvement), bilateral tubes and ovaries (*in situ*)	12	NED
Marwah *et al.* (2012) [14]	3	65	Pyometra	Invasive	Endometrium (*in situ*)	NA	NA
		60	Vaginal bleeding	Invasive	Endometrium (*in situ* with small focal microinvasion)	NA	NA
		49	Vaginal bleeding	Invasive	Endometrium (*in situ*)	NA	NA
Chao *et al.* (2013) [4]	1	60	Pyometra	Carcinoma *in situ*	Endometrium (*in situ*, foci microinvasive)	–	DOD (2 days)
Ishida *et al.* (2013) [5]	2	64	Vaginal bleeding	Invasive	Endometrium (*in situ*)	NA	NA
		59	Vaginal bleeding	Invasive	Endometrium (*in situ*)	NA	NA
Yang *et al.* (2014) [37]	1	69	Hydrometra	Carcinoma *in situ*	Uterine corpus, vagina, left salpinx (all *in situ* with multifocal microinvsive)	NA	NA
Anthuenis *et al.* (2016) [38]	1	72	Hydrometra	Carcinoma *in situ*	Endometrium (*in situ*, focal microinvasive)	24	NED
Neelam *et al.* (2017) [39]	2	60	Abdominal mass	Invasive	Endometrium (*in situ*)	NA	NA
		70	Abdominal mass	Carcinoma *in situ*	Endometrium (*in situ*)	NA	NA
Muthusamy *et al.* (2017) [40]	1	45	Vaginal bleeding, lower abdominal pain	Carcinoma *in situ*	Endometrium (*in situ*)	NA	NA
Nakajima *et al.* (2019) [2]	1	67	Lower abdominal pain	Carcinoma *in situ*	Endometrium (*in situ*), bilateral tubes (*in situ*), both ovaries (invasive), greater omentum (invasive)		
Du *et al.* (2019) [41]	1	66	Abnormal pap smears	Carcinoma *in situ*	Endometrium (*in situ*, foci microinvasive)	43	NED
Current study	1	66	Abnormal pap smears, pyometra	Invasive	Endometrium (*in situ*)	6	NED

*SCC* squamous cell carcinoma, *NA* not available, *NED* no evidence of disease, *DOD* died of disease

Carcinoma *in situ* (preinvasive or CIN III) or invasive carcinoma that is strictly confined to the cervix or extends into the uterine corpus (stage I, FIGO 2018) is by definition a histologic diagnosis. Nevertheless, cervical SCC with superficial spread to the endometrium or upper genital tract is not recognized in the latest FIGO or WHO classification systems. The main site affected by superficially spreading cervical SCC in the cases reported to date has been the endometrium, generally in isolation and without signs of invasive growth (carcinoma *in situ*). There have, however, been a few reports of unilateral or bilateral involvement of the fallopian tubes and/or ovaries in addition to endometrial extension. There has just been one report of distant metastasis, involving the greater omentum [2].

A genetic study of five patients with superficial spreading cervical SCC showed a single clonal process and frequent loss of heterozygosity at 6p, 6q, 11p, and 11q [3], all loci that are typically lost in cervical SCC. Consistent with our case, superficial spreading cervical SCC stains positively for p16, a surrogate immunohistochemical marker of HPV. HR-HPV infection is known to have a key pathogenic role in cervical SCC [4]. In one of the studies reviewed, all the samples analyzed were HPV 16 positive [4], suggesting that persistent HR-HPV infection is a key factor in the development of superficial spreading cervical SCC. In another study, CD138 was strongly expressed in superficial carcinoma cells in both the cervix and endometrium [5], suggesting that it may also be involved through the regulation of cell–cell interactions.

Conization followed by regular cytology and HPV detection (co-testing) is the standard procedure for CIN III management and follow-up. Cervical stenosis is a late complication of conization [6] and can result in unsatisfactory cytological and colposcopy follow-up and consequently higher false-negative rates and fewer early detections of recurrence [2]. Apart from cervical and vaginal cytology, patients with post-conization cervical stenosis should undergo additional procedures such as endocervical cytology, endometrial biopsy, and/or transvaginal pelvic ultrasound, especially if they have persistent HR-HPV infection.

As superficial spreading SCC of the cervix is so rare, there is limited information on its prognosis or clinical management [7–9]. It is more common in postmenopausal women, and the main presenting signs are vaginal bleeding and discharge (Table 1). HPV genotyping in combination with cervical and vaginal cytology is useful. An important role for local immune intolerance has been postulated. HPV vaccination is probably the only primary prevention measure possible. There have been reports that superficially spreading cervical SCC with endometrial involvement has worse prognosis than standard endometrial SCC [10–13]. Tumor volume and lymphovascular invasion are known risk factors for recurrence in cervical cancer and are also predictive of lymph node metastasis. Cervical stenosis with pyometra [14] and previous radiotherapy [15] can favor superficial spread. It is currently difficult to draw any conclusions regarding optimal treatment. Based on FIGO 2018 recommendations, a simple hysterectomy would be sufficient for SCC *in situ* or stage IA1 SCC without nodal involvement and an isolated focus of carcinoma *in situ* in the endometrium.

SCC of cervix is the most common tumor of the female genital tract, accounting for approximately 80% of all cervical cancers. Carcinoma of the cervix generally spreads upwards to the parametrium and through lymphatic invasion to the uterine wall. Although superficial spreading SCC of the cervix is rare, it should be considered in postmenopausal women with past history of cervical conization and persistent HR-HPV infection, as early diagnosis is important. There are insufficient data to compare superficial spreading SCC of the cervix with other types of cervical cancer. More clinical cases are needed to identify additional prognostic factors and inform clinical practice guidelines on the management of this disease.

## Data Availability

Not applicable.

## Abbreviations

CIN: Carcinoma *in situ*
CRP: C-reactive protein
FIGO: International federation of gynecology and obstetrics
HIV: Human immunodeficiency virus
HR-HPV: Human high-risk papillomavirus
SCC: Squamous cell carcinoma
WHO: World health organization
